# Barriers to prevention and healthcare-seeking in a fragile humanitarian setting: Evidence from Yemen during the COVID-19 pandemic

**DOI:** 10.1371/journal.pgph.0007435

**Published:** 2026-10-15

**Authors:** Ola El Hajj Hassan, Ahmed Asa'ad Al-Aghbari, Huda Omer Basaleem, Khaled Abdulla Al-Sakkaf, Dalia Hyzam, Ahmad Siyar Noormal, Albrecht Jahn, Fekri Dureab

**Affiliations:** 1 Heidelberg Institute of Global Health, Heidelberg University Hospital, Heidelberg, Germany; 2 Faculty of Medicine and Health Sciences, University of Aden, Aden, Yemen; 3 Institute of Research for International Assistance, Akkon Hochschule, Berlin, Germany; 4 Department of Healthcare Management, University of Doha for Science and Technology, Doha, Qatar; Johns Hopkins University Bloomberg School of Public Health, UNITED STATES OF AMERICA

## Abstract

The healthcare system in Yemen is confronted with substantial challenges, especially during pandemics such as COVID-19. In Aden, the COVID-19 pandemic occurred in the context of prolonged conflict, health-system fragility, resource constraints and other ongoing humanitarian challenges.This study aims to explore the barriers community members in Aden face in adopting COVID-19 preventive measures and seeking healthcare during the pandemic, and to identify implications for future outbreak preparedness in fragile humanitarian settings. A qualitative study involving 25 in-depth interviews was conducted with adult community members in Aden. Participants’ perceptions of COVID-19 and reported preventive practices were also explored to contextualise the barriers influencing prevention and healthcare-seeking. Interview data were analysed using inductive qualitative content analysis; the Socio-Ecological Model (SEM) was applied as an interpretive framework to contextualise barriers across five interacting levels: intrapersonal/individual, interpersonal, organisational/institutional, community, and public policy/structural. Participants described barriers across five SEM levels: intrapersonal/individual barriers included denial, fear, perceived ineffectiveness of preventive measures and financial constraints; interpersonal barriers included stigma, mockery and social pressure; organisational/institutional barriers included perceived service refusal, concerns about infection prevention and staff preparedness, long waiting times, limited diagnostic testing, increased treatment costs and shortages of medical supplies; community-level barriers included rumours, misinformation and mistrust; and public policy/structural barriers included limited financial support, inadequate implementation of preventive measures, conflict-related constraints and perceived insufficient preparedness. The findings suggest that barriers to prevention and healthcare-seeking were interconnected across the five levels of the socio-ecological context, with individual perceptions and behaviours occurring within interpersonal, organisational, community and wider public policy and structural conditions. Future outbreak preparedness in fragile settings should therefore combine trusted, culturally appropriate risk communication, community engagement, accessible preventive resources, strengthened infection-prevention capacity, diagnostic and referral pathways, and continuity of essential health services.

## Introduction

Coronavirus disease (COVID-19) was declared a public health emergency of international concern on 30 January 2020 and characterised as a global pandemic on 11 March 2020 [1]. As of October 2023, more than 771 million confirmed COVID-19 cases and nearly 7 million deaths had been reported globally [2]. Early in the pandemic, global health authorities warned of the virus's potential for rapid, severe spread, especially in low- and middle-income countries where healthcare infrastructure is fragile, health capacity is limited, and access to medical resources is insufficient [3]. These challenges were severe in Yemen, where the health system had already been weakened by years of conflict, widespread displacement, natural disasters, and recurring disease outbreaks such as cholera, diphtheria, and measles [3,4]. In total, 20 out of Yemen's 22 Governorates have been severely affected by conflict, with civilians bearing the brunt of the violence [3]. An early 2016 regional overview by the United Nations Office for the Coordination of Humanitarian Affairs (UN OCHA) revealed that Yemen accounted for 37% of all those classified as in humanitarian need regionally, equivalent to 21.2 million or 82% of the Yemeni population [3]. Almost 54% of the population lacks access to healthcare, ranking Yemen as the second-highest country for this index in the Middle East [3,4]. Moreover, only half of hospitals and health facilities are functional, and even those operating function below their optimal capacity [5]. In addition, Yemen has only 10 health workers per 10,000 people, indicating a substantial shortage of healthcare personnel. The shortage of health personnel was exacerbated by the suspension of salaries for approximately 1.5 million state employees, including health workers [6]. This challenging situation hinders the ability of the healthcare system to deliver adequate and effective medical services, affecting both healthcare professionals and individuals seeking medical care [7]. On top of all these factors, COVID-19 added to the health system’s struggle to meet people's healthcare needs, with only one operational hospital in the city of Aden, highlighting the immense challenges and vulnerabilities faced at the height of the pandemic [7]. As a result, communities in Yemen had to navigate overlapping challenges, including hunger, infectious diseases, conflict, displacement, and COVID-19 [7].

All these preexisting adversities, together with the pandemic, created a complex humanitarian crisis in which people had to navigate multiple health and socioeconomic pressures. Although barriers to COVID-19 prevention were documented in different settings, little is known about how community perceptions, health-system weaknesses, and broader structural conditions interact to influence both preventive behaviour and healthcare seeking in conflict-affected Yemen.

To support interpretation of these interacting influences, this study draws on the Socio-Ecological Model (SEM) as a conceptual framework [8,9]. The SEM recognises that health behaviours are shaped by influences operating at multiple and interacting levels [8]. For this study, the framework was operationalised across five levels: intrapersonal/individual factors, including perceptions, beliefs and individual capacity to adopt preventive actions; interpersonal influences arising through social relationships and interactions; organisational/institutional factors related primarily to healthcare facilities and service delivery; community-level influences, including shared norms, rumours, misinformation and mistrust; and public policy/structural factors, including governmental preparedness, resource constraints and the broader effects of prolonged conflict.

Therefore, this study aimed to explore community members’ experiences and perceptions of barriers to COVID-19 prevention and healthcare seeking in Aden, Yemen, and to examine how barriers operating across multiple levels of the socio-ecological context interacted during the pandemic. The findings are interpreted through the SEM to identify context-relevant implications for future public health emergencies in fragile and conflict-affected settings.

## Materials and methods

### Ethics statement

The study involving human participants was reviewed and approved by the Ethical Review Committee of Heidelberg University (protocol code: S-887/2020 and date of approval: 15 February 2021) and by the Ethical Review Committee of the University of Aden (protocol code: REC-96–2021 and date of approval: 23 March 2021). All participants provided their written informed consent to participate in this study.

### Study setting

This qualitative study was conducted in Aden governorate in Yemen. Aden consists of eight districts: Sira, Khormaksar, Al Mualla, Al Tawahi, Sheikh Othman, Mansoura, Dar Saad, and Brega. Aden was selected as an important urban setting affected by the COVID-19 pandemic and provided an important setting for exploring community experiences of COVID-19 prevention and healthcare seeking within a health system already affected by prolonged conflict and resource constraints.

### Study population and sampling

The study population comprised adult residents of Aden governorate. In-depth interviews (S1 Appendix) were conducted with participants to investigate their experiences of COVID-19 prevention and healthcare-seeking and the barriers influencing these behaviours during the pandemic. Participants were selected from a larger sample of 400 individuals who had previously completed a community-based COVID-19 questionnaire as part of the wider knowledge, attitudes, and practices (KAP) study conducted in Aden [7]. The survey participant list served as the sampling frame. To initiate the systematic sampling procedure, one participant was randomly selected from among the first 16 participants on the survey list. Thereafter, every 16th participant on the list was selected and invited to participate in an in-depth interview. This systematic procedure provided a structured approach to participant selection and was intended to capture a range of perspectives from the parent survey sample rather than to achieve statistical representativeness. All 25 invited participants agreed to participate; therefore, no refusals occurred, and no replacements were required.

A total of 25 interviews were planned and conducted. Data collection and preliminary analysis overlapped, allowing the research team to review emerging codes, themes, and new information as the interviews progressed. Saturation was assessed during concurrent data collection and preliminary analysis by the principal investigator and two researchers from Heidelberg University. Data saturation was considered to have been reached at the 22nd interview, when no new themes, insights, or substantively new information relevant to the study objectives emerged. The remaining three interviews were completed as planned and did not generate additional themes. All 25 interviews were transcribed and included in the final qualitative analysis. Eligible participants were aged 18 years or older, had no symptoms of COVID-19, were not isolated or in quarantine at the time of data collection, and were residents of Aden governorate.

### Data collection tools and methods

Data collection was carried out by two research teams, each comprising one research professional and two interviewers, between 24 March 2021 and 30 May 2021. Face-to-face semi-structured interviews were conducted at the participants’ households. Before data collection, interviewers received training in qualitative interviewing techniques, including establishing rapport, using open-ended questions, allowing participants to describe their experiences in their own words, and maintaining neutrality to minimise leading questions and interviewer influence on participants’ responses.

Initially, all participants received the study's information sheet and consent form and were allowed to review the study information before providing written informed consent. Upon obtaining participants’ consent, twenty-five uninterrupted face-to-face interviews were conducted. Interviews lasted approximately 25–35 minutes and were audio recorded.

Researchers used an in-depth interview (IDI) guide specifically developed for this study (S1 Appendix). The guide explored participants’ perceptions and beliefs regarding COVID-19, preventive practices, community-level barriers, experiences and perceptions of healthcare services, governmental and structural barriers, and factors affecting healthcare-seeking decisions. Participants’ perceptions of COVID-19 and their reported preventive practices were examined because they provided contextual information for understanding how the identified barriers influenced preventive behaviour and decisions to seek healthcare. The semi-structured format also allowed interviewers to use follow-up probes to clarify responses and explore issues raised by participants in greater depth. Before data collection, the guide was reviewed by the research team, four university researchers, one German international health expert, three Yemeni researchers, and two medical doctors. It was then pilot-tested with community members who had sociodemographic characteristics similar to those of the study population. Two questions were revised to improve their clarity and sequencing before formal data collection. The core domains of the interview guide remained unchanged during data collection, although interviewers used additional probes where necessary to explore emerging issues.

### Transcription and translation

Interviews were conducted in Arabic, and the audio recordings were transcribed verbatim in Arabic and then translated into English by bilingual members of the research team. The translated transcripts were reviewed against the original Arabic transcripts and, where necessary, the audio recordings, to clarify ambiguous wording and preserve the intended meaning of participants’ accounts. English-language transcripts were subsequently used for analysis. Both the interview audio recordings and interview notes were securely stored.

### Data analysis

The interview data were analysed using an inductive qualitative content analysis approach. This approach enabled the systematic identification, organisation, and interpretation of patterns within the interview transcripts [10,11]. Data analysis began during the early stages of data collection. Following transcription and translation, the verbatim transcripts were reviewed by two research professionals and two principal researchers to check the accuracy of the transcription and translation. The English-language transcripts were imported into NVivo 12 software for data management and inductive coding. Transcripts were examined line by line and paragraph by paragraph to generate initial codes.

After this step, similar codes were grouped into sub-themes and then into overarching themes. Initial coding and theme development were undertaken independently by two research professionals. The emerging codes, sub-themes, and overarching themes were subsequently reviewed by two principal researchers. Differences in coding or interpretation were discussed among the researchers until consensus was reached, and the final thematic structure was agreed upon by the research team.

Later, an analytic coding tree was developed to organise the relationships between codes, sub-themes, and overarching themes in relation to the study objectives. All data within each theme were reviewed to ensure that the themes reflected the range of participants’ accounts. Representative participant quotations were selected to illustrate the principal findings.

The initial coding process was inductive. Following development of the themes, the Socio-Ecological Model was used as an interpretive framework to examine how the identified barriers operated and interacted across five levels: intrapersonal/individual, interpersonal, organisational/institutional, community, and public policy/structural. The inductively developed themes were subsequently mapped to these levels according to the primary context in which each barrier operated. Because several barriers extended across more than one level, the SEM categories were not treated as mutually exclusive. The framework was therefore used to support the organisation and interpretation of the inductively generated findings rather than to impose predetermined categories during the initial coding process.

### Research team and reflexivity

The research team included researchers based in Yemen and at international academic institutions. The Yemeni researchers contributed knowledge of the local language, culture, and health system, while the wider team contributed public health and qualitative research experience. Reflexive discussions were held during data collection and analysis. The team discussed how their professional backgrounds and knowledge of the Yemeni context might influence data collection and interpretation. Emerging findings were reviewed by several researchers, and differences in interpretation were discussed within the team to challenge assumptions and strengthen the interpretation of the findings.

### Trustworthiness

Several procedures were used to enhance the credibility and trustworthiness of the findings. Data collection and analysis overlapped, allowing emerging information to be reviewed as interviews progressed and supporting the assessment of data saturation. Multiple researchers participated in coding and review of the emerging thematic structure. Differences in interpretation were discussed within the research team and resolved through consensus. During the interviews, participants were also given opportunities to clarify or confirm the interviewer's understanding of their responses, helping to ensure that their intended meanings were accurately understood. The reporting of this qualitative study followed the Consolidated Criteria for Reporting Qualitative Research (COREQ) [12] (S2 Checklist).

### Inclusivity in global research

Additional information regarding inclusivity in global research is provided in the Supporting Information (S1 Checklist).

## Results

### Characteristics of the participants

A total of 25 participants were included in the final qualitative analysis. Their sociodemographic information is summarised in Table 1. The sample included 14 women and 11 men, with a mean age of 36.8 years (SD 11.1, range 19–66).

**Table 1:** Sociodemographic characteristics of participants included in the qualitative interviews (n = 25).

	Category	n (%)
Age, years	Mean (SD) Range (19–66 years)	36.8 (11.1)
Sex	Female	14 (56.0)
	Male	11 (44.0)
Marital status	Married	18 (72.0)
	Other	7 (28.0)
Education	Up to middle school	3 (12.0)
	High school/university	22 (88.0)
Occupation	Employed	13 (52.0)
	Not employed	12 (48.0)

Note: “Not employed” includes students, retired participants, and those reporting no employment. Education categories were combined to provide a concise description of the qualitative sample.

### Contextual findings: Perceptions and preventive practices

Participants’ perceptions of COVID-19 and their reported preventive practices are presented first to provide context for understanding the barriers described subsequently. These findings illustrate how participants understood the disease and how these understandings were reflected in reported preventive behaviour before the barriers were interpreted within the SEM.

### Perceptions and beliefs about COVID-19

Participants described initial disbelief following the emergence of COVID-19 in Yemen, even though health authorities had confirmed its presence. Several participants described initial uncertainty regarding the disease and preventive measures, while others reported that recognition of COVID-19 increased as cases and deaths became more visible.

*“Initially, the existence of COVID-19 was denied until the Ministry of Health established specific health facilities and centres to deal with COVID-19. At first, our neighbours referred to it as ‘Jazaa,’ [flu] and people did not promptly adopt precautionary measures.”* (Informant 1)

“*Initially, people denied the existence of the epidemic, despite health authorities announcing its presence.” (*Informant 5)

“At *the beginning, people were unwilling to accept the existence of this disease and continued to live with it as part of their normal life*” (Informant 20)

As more information about COVID-19 and preventive measures became available, some participants described increasing awareness. However, participants’ accounts also indicated continued uncertainty, inconsistent adoption of preventive measures, and fear of infection. These accounts suggest that perceptions of COVID-19 were not static but changed as participants gained information and observed the effects of the pandemic within their communities.

“*At the beginning of the pandemic, the COVID-19 topic was unclear. Then I got more information from the media. People did not take necessary precautionary measures; you know why? Whoever wore a mask at that time was accused of having COVID-19, even though it was a prevention measure*.” (Informant 1)

Some participants used the local term “Jazaa” to describe what they perceived as a mild flu-like or seasonal illness and distinguished it from COVID-19. Their accounts suggested that using a familiar illness label may have reduced the perceived seriousness of symptoms or distanced their experience from the more threatening concept of COVID-19.

“*I don't mind visiting someone with this Jazaa. COVID-19 is exaggerated in the media.”* (Informant 19)

“*I didn't have COVID-19; it was just Jazaa.”* (Informant 22)

Some participants also described a change in perceptions as the pandemic progressed, reporting reduced fear and a perception that the situation had improved.

“*No one is dying from COVID-19 anymore.”* (Informant 1)

“*The current state of affairs concerning COVID-19 has shown improvement compared to the initial stages of the pandemic, with a decline in the number of cases*.” (Informant 22)

### Preventive practices reported by participants

Participants described varied approaches to COVID-19 prevention. Some individuals highlighted their proactive efforts to protect themselves and others during the pandemic. These efforts include adhering to precautions such as wearing masks, washing hands regularly, maintaining social distancing (1.5 meters) and avoiding gatherings, including funerals and weddings. These accounts demonstrate variation in preventive behaviour among participants rather than a uniform community response.

“I *took precautions, including wearing a mask, practising handwashing, maintaining social distancing, and avoiding any gatherings such as funerals or weddings. However, I had to purchase masks for myself*.” (Informant 4)

"*I practised precautions, including social distancing and avoiding sharing utensils for drinking water. Additionally, I consumed most of my meals at home and wore a mask when in the presence of other people*." (Informant 7)

"*The preventive measures I have implemented include refraining from touching surfaces. In case of contact, I ensured to wash my hands with disinfectants and soap while also practising social distancing*.” (Informant 14)

In contrast, other participants questioned the effectiveness or feasibility of recommended preventive measures and vaccination. Some described relying on home remedies such as ginger, steam inhalation or herbal products, while others reported taking few or no preventive actions. These contrasting accounts indicate that knowledge of recommended measures did not necessarily result in their adoption.

“*I do not believe that practices such as handwashing, wearing masks, and maintaining hygiene are preventive and effective. I also think that social distancing is not applicable in our society. Even in health facilities, no one is wearing a mask*.” (Informant 2)

“*When I observed many people dying despite taking precautions, I discontinued my preventive measures."* (Informant 3)

"*I don't believe that precautionary measures are effective. If someone wants to get COVID-19, they will have it despite whatever they do*." (Informant 19)

"*I believe that home-based treatment is superior, and the utilisation of natural herbs such as black seed, ginger, and cloves eliminates the need to visit the hospital." (*Informant 22)

### Barriers to COVID-19 prevention and healthcare-seeking across the Socio-ecological model

The inductively identified barriers were subsequently interpreted using the SEM. They are presented below in the order of the five SEM levels: intrapersonal/individual, interpersonal, organisational/institutional, community, and public policy/structural. The classification indicates the primary level at which each barrier operated; participants’ accounts also showed substantial interaction across levels.

### Intrapersonal/Individual level

At the intrapersonal/individual level, participants described barriers related to their own perceptions, emotional responses and ability to adopt preventive behaviours. These included denial or uncertainty regarding the existence or severity of COVID-19, fear of infection, perceived ineffectiveness or limited feasibility of recommended preventive measures, and difficulty affording preventive supplies or healthcare-related costs. These factors influenced how participants perceived the risks associated with COVID-19 and whether preventive measures and formal healthcare were considered necessary or feasible. Examples are presented in Table 2.

**Table 2:** Participant-reported intrapersonal/individual and interpersonal barriers to COVID-19 prevention and healthcare-seeking in Aden, Yemen.

Primary SEM level	Theme	Description	Representative quote
Intrapersonal/individual	Denial	Participants’ denial or uncertainty regarding the existence/severity of COVID-19	“People in society do not accept the idea of the existence of this disease.” (Informant 9)
Intrapersonal/individual	Fear	Fear of infection, including fear of acquiring infection in healthcare facilities	“I am afraid to get infected in health facilities because they do not adhere to any preventive measures.” (Informant 1)
Intrapersonal/individual	Perceived ineffectiveness or limited feasibility of preventive measures	Participants questioned the effectiveness or feasibility of recommended preventive measures	“I don't believe that precautionary measures are effective. If someone wants to get COVID-19, they will have it despite whatever they do.” (Informant 19)
Intrapersonal/individual	Financial constraints	Difficulty affording preventive supplies and healthcare-related costs	“We had to buy the masks for ourselves, and it was hard.” (Informant 4)
Interpersonal	Stigma	Perceived stigma associated with infection and visible preventive behaviour	“Whoever wears a mask was accused of having COVID disease, even though it is a prevention measure.” (Informant 1)
Interpersonal	Mockery and social pressure	Mockery or social pressure directed towards individuals adopting preventive measures	“If I wear a mask, they will laugh at me and make jokes.” (Informant 1)

### Interpersonal-level barriers

At the interpersonal level, participants described stigma, mockery and social pressure associated with COVID-19 and with visible preventive behaviours. Participants reported that individuals wearing masks could be perceived as infected and that people adopting preventive measures could be laughed at or mocked. These social reactions could make adherence to visible preventive measures socially difficult despite awareness of their intended protective role. Examples are presented in Table 2.

### Perceived Organisational/institutional-level health-system barriers

Participants reported several organisational and institutional health-system factors that influenced their confidence in and use of healthcare services during the pandemic. These included perceived refusal of services in some facilities, concerns about infection-prevention practices and staff preparedness, long waiting times, limited access to PCR testing, increased treatment costs and shortages of medical supplies. Within the SEM, these barriers were interpreted primarily at the organisational/institutional level because they concerned the functioning, capacity and delivery of services within healthcare organisations. These accounts describe participants’ experiences and perceptions and should not be interpreted as an assessment of all healthcare facilities in Aden. Participants frequently described these service-related barriers alongside fear and mistrust, suggesting that perceived weaknesses in service delivery could reinforce reluctance to seek formal healthcare. Representative quotations are provided in Table 3.

**Table 3:** Participant-reported organisational/institutional health-system barriers affecting COVID-19 prevention and healthcare-seeking in Aden, Yemen.

Theme	Description	Illustrative quote
Perceived refusal of service provision	Participants reported that some healthcare facilities initially refused to receive patients, including suspected COVID-19 cases.	“At health care services, they did not accept to receive cases at first.” (Informant 3)
Concerns about quality of care and infection prevention	Participants reported concerns regarding infection-prevention practices and interactions with healthcare workers.	“Even in health facilities, no one is wearing a mask.” (Informant 2)
Perceived limited staff knowledge and experience	Some participants perceived limited knowledge of or experience among healthcare staff in managing COVID-19.	“There is a lack of knowledge and experience of the disease from the medical staff.” (Informant 21)
Long waiting times	Participants reported that long waiting times discouraged use of healthcare services.	“Health facility had a long waiting time of 3–7 hours; that is why I prefer to treat myself at home.” (Informant 2)
Limited access to diagnostic testing	Participants reported limited access to PCR testing.	“There is a lack of PCR testing in all hospitals.” (Informant 9)
Increased treatment costs	Participants reported increased costs of treatment and private healthcare services during the pandemic.	“Increase in the cost of expensive treatments and private hospitals.” (Informant 14)
Perceived shortages of medical supplies	Participants perceived shortages of essential medical supplies and resources in health facilities.	“They do not have any medical supplies, so they do not give their best treatment and services.” (Informant 4)

Table note: PCR = polymerase chain reaction

### Community-level barriers

At the community level, participants described circulating rumours and misinformation concerning COVID-19 and healthcare facilities, together with broader mistrust in public-health authorities and aspects of the health response. These shared narratives and perceptions extended beyond individual beliefs and were described as circulating within the wider social environment, potentially reinforcing scepticism, fear and reluctance to seek formal healthcare. Participants also described mistrust concerning aspects of the health response and vaccine handling. Mistrust therefore intersected with individual risk perceptions and confidence in health institutions, illustrating that some barriers operated across more than one SEM level.

*“If you go to the health facility, they will isolate you and give you injections to kill you.”* (Informant 9)

*“I did not take the vaccine because I believe it is not well stored at a good temperature in our country.”* (Informant 9)

### Public policy/structural-level barriers

Participants perceived several public policy and structural factors as barriers to an effective COVID-19 response. These included limited financial support, inadequate implementation of preventive measures, conflict-related constraints and shortages of medical resources. Several participants also expressed concerns regarding governmental preparedness for pandemics and other public-health emergencies. These findings represent participants’ perceptions of the response rather than an independent assessment of government performance. Participants’ accounts indicated that these wider structural conditions also affected their ability to access preventive resources and contributed to pressures on the health system.Within the SEM, these barriers were interpreted primarily at the public policy/structural level because they reflected wider governmental, economic and conflict-related conditions shaping the resources and institutional capacity available for outbreak response.

“...*We had to buy masks on our own, and not everyone could afford them. The healthcare system faced shortages, possibly due to the ongoing war. In 2020, we were in the fifth year of conflict, with the situation neither fully at war nor peace, as the government grapples with managing the challenges. Despite the government posting guidelines on how to protect and avoid infection, there were occasional market closures enforced*.” (Informant 4)

“...*The government did not enforce strict precautions; only a few people wore masks, and it was not mandatory.”* (Informant 9)

"…*The government's preventive measures aimed at raising awareness and minimising risks were insufficient. However, they did not offer financial support to the public or provide medicines and medical devices."* (Informant 14)

### Interconnections across levels

Taken together, participants’ accounts indicated that barriers to prevention and healthcare-seeking operated across all five SEM levels and did not occur in isolation. At the intrapersonal/individual level, beliefs about COVID-19, fear, perceived effectiveness of preventive measures and financial capacity influenced individual responses. At the interpersonal level, stigma, mockery and social pressure affected the social acceptability of preventive behaviours. Organisational/institutional barriers influenced participants’ confidence in and access to formal healthcare, while community-level misinformation and mistrust shaped perceptions of both COVID-19 and the health response. Public policy and structural conditions, including conflict-related constraints, resource limitations and perceived inadequate preparedness, influenced the environment within which communities and health services responded. Viewed through the SEM, these findings suggest that barriers at one level could reinforce those experienced at other levels rather than operating independently [8,9].

## Discussion

This study identified interacting barriers to COVID-19 prevention and healthcare seeking across the five levels of the SEM in Aden: intrapersonal/individual, interpersonal, organisational/institutional, community and public policy/structural. At the intrapersonal/individual level, beliefs about COVID-19, fear, perceived effectiveness of preventive measures and financial capacity influenced reported behaviour. Interpersonal influences included stigma, mockery and social pressure; organisational/institutional barriers concerned healthcare access, preparedness and service capacity; community-level barriers included misinformation and mistrust; and public policy/structural barriers reflected governmental preparedness, resource constraints and prolonged conflict. Interpreted through the Socio-Ecological Model, the findings suggest that preventive behaviour and decisions to seek healthcare were not determined solely by individual knowledge or attitudes. Rather, they occurred within a wider environment including social pressure, misinformation, mistrust, financial constraints, perceived limitations in healthcare delivery and the effects of prolonged conflict on institutional capacity [8,9,13,14]. This multi-level interpretation is particularly relevant in a fragile setting such as Yemen, where community behaviour is shaped by the interaction between individual perceptions, social relationships, health-system capacity and wider structural instability [13].

The findings of this study showed that some participants initially expressed disbelief and uncertainty regarding COVID-19 despite official confirmation of the disease. As the pandemic progressed, participants described greater recognition of the disease, but this did not necessarily translate into consistent preventive behaviour.

This finding is consistent with the previous quantitative study conducted in Aden, which showed a gap between knowledge of COVID-19 prevention and actual adoption of preventive practices [7]. The present qualitative findings help explain this knowledge–practice gap by showing that preventive behaviour was influenced not only by information but also by factors operating across different socio-ecological levels, including social stigma, perceived ineffectiveness of preventive measures, misinformation, mistrust and financial constraints. Thus, the qualitative findings provide contextual explanations for patterns previously observed quantitatively. The qualitative component therefore adds an important explanatory dimension to the earlier quantitative findings: knowing recommended preventive measures did not necessarily mean that participants considered those measures socially acceptable, affordable, effective or feasible within their everyday circumstances.

### The cultural meaning of “Jazaa”

A particularly important finding was participants’ use of the local term “Jazaa.” Rather than functioning only as an alternative name for COVID-19, participants’ accounts suggest that the term may have provided a familiar cultural interpretation for an unfamiliar and threatening illness. Describing symptoms as “Jazaa,” a relatively familiar flu-like condition, may have normalized the illness and reduced perceived severity. In this sense, the term may have provided a way for some individuals to interpret COVID-19 in less threatening terms. This interpretation should be treated cautiously because the study was not specifically designed to investigate illness narratives. Nevertheless, the finding has important implications for outbreak communication. Risk communication in similar settings should not rely only on biomedical terminology but may benefit from identifying and engaging with locally used illness concepts. Understanding how communities name and interpret emerging diseases may help public-health actors develop messages that acknowledge familiar explanations while clearly communicating new risks and recommended actions [15,16].

### Divergent experiences and preventive behaviour

Some participants demonstrated proactive efforts to protect themselves and others by following measures such as mask-wearing, handwashing, social distancing and avoiding gatherings. Other participants, however, questioned the effectiveness of these practices or relied on home-based remedies. These contrasting experiences are important because they demonstrate that the study population was not homogeneous. Exposure to the same outbreak produced different interpretations and behavioural responses.

The coexistence of precautionary behaviour and scepticism also suggests that low preventive adherence cannot be attributed simply to lack of knowledge. Participants’ descriptions indicate that perceived effectiveness, social acceptability, trust, affordability and confidence in health institutions shaped whether recommended behaviours were considered feasible or worthwhile [7,17,18].

### Interactions across the five levels of the Socio-ecological model

One of the central findings of this study is that barriers at different SEM levels appeared to reinforce one another. Participants’ accounts suggest interconnections whereby chronic health-system fragility and perceived governmental unpreparedness contributed to reduced confidence in the health response. Limited confidence in institutions and health services may, in turn, have increased the influence of rumours and misinformation. Such misinformation could reinforce denial, fear and stigma, while negative perceptions of healthcare facilities discouraged some individuals from seeking formal care.

These relationships should not be interpreted as causal pathways established by this qualitative study. Rather, they illustrate how participants experienced different barriers as interconnected. From a socio-ecological perspective, this is important because an intervention targeting only one level, such as individual knowledge, would be unlikely to overcome simultaneous barriers related to interpersonal influences, community trust, affordability, social norms, organizational capacity and wider structural constraints.

In this context, prolonged conflict can be understood as a structural condition that may have intensified several downstream barriers [13–15]. Conflict-related disruption and resource constraints affected health-system capacity and preparedness; perceived weaknesses in the health response were associated with mistrust; and mistrust may have created an environment in which rumours and misinformation could become more credible. These community-level perceptions could then influence preventive behaviour and willingness to seek healthcare. The findings therefore suggest that the effects of COVID-19 cannot be considered separately from the chronic fragility within which the pandemic occurred [13,14,19].

Social stigma has harmful effects on individuals and communities, distorting perceptions of risk and worsening the epidemic situation [20,21]. In the present study, stigma extended beyond infection itself: participants reported that mask wearing could also become socially stigmatising because it was interpreted as evidence that the person might have COVID-19. Preventive behaviour could therefore carry a social cost. This finding shows that a recommended preventive behaviour could paradoxically identify an individual as potentially infected and expose them to negative social reactions. In such circumstances, adherence depends not only on understanding health recommendations but also on whether individuals are willing and able to accept their social consequences.

Moreover, misinformation about healthcare facilities and the negative perceptions of service provision was described as factors discouraging healthcare-seeking. Participants’ accounts also suggested that misinformation could reinforce disbelief and social pressure surrounding preventive measures. These findings are consistent with the authors’ previous mixed-methods work in Aden, which identified an association between misinformation, scepticism and preventive behaviour [17].

Together, the findings suggest a reinforcing relationship: mistrust may increase the plausibility of rumours; rumours may reinforce fear and scepticism; and fear and scepticism could further reduce engagement with health services and official recommendations. Conversely, negative experiences or perceptions of healthcare services could reinforce existing rumours and mistrust. This reciprocal relationship between institutional trust, misinformation and healthcare seeking is important for preparedness because communication interventions alone may have limited impact if communities continue to perceive health services as inaccessible, unsafe or poorly prepared.

### Fragile and conflict-affected settings

Challenges such as misinformation, stigma, fear and inconsistent adoption of preventive practices have also been reported in other low- and middle-income settings. Evidence across LMICs similarly shows that financial barriers, difficulties accessing services, misinformation and mistrust can affect healthcare use and preventive behaviour even in settings not directly affected by conflict [18,22]. These barriers should therefore not be considered specific to Yemen or to conflict-affected settings. However, the findings from Aden suggest that these commonly observed barriers were intensified by conditions characteristic of a prolonged humanitarian crisis, including weakened health infrastructure, shortages of medical resources, constrained institutional capacity and financial barriers to accessing services [13,14,19].

The distinction between a generally resource-constrained setting and a fragile or conflict-affected setting is important. In Yemen, pandemic-related challenges were superimposed on years of conflict, disruption of health services, workforce shortages and weakened governance [13,19]. Consequently, barriers such as inability to afford preventive supplies, limited diagnostic capacity and mistrust in institutions did not emerge solely because of COVID-19; they reflected and magnified pre-existing structural weaknesses. A recent review of systematic reviews in fragile and conflict-affected states similarly identified health-workforce constraints, limited training capacity and resources, and the importance of coordinated and context-adapted health-system responses [14]. Evidence from Afghanistan during and after COVID-19 also showed that the pandemic compounded an already fragile health system, with persistent service backlogs, increased costs and limited availability of medicines affecting access to essential healthcare [23].

Thus, in the present study, misinformation, stigma, fear and financial barriers can be understood as challenges also found across LMICs, while prolonged service disruption, shortages of health-system resources, weakened institutional capacity and limited emergency preparedness were amplified by Yemen’s conflict-affected context. Conflict therefore did not necessarily create each individual barrier but increased their severity and made them more difficult to address.

Furthermore, challenges at the governmental level raised in this study included a lack of financial support for communities, inadequate prevention measures, the effects of war, and the inability to provide essential medical resources. Concerns were also expressed by participants regarding the government's readiness to address these issues. Yemen's healthcare system is fragile and has been severely weakened due to long periods of conflict and weakened governance [13,19]. Since the beginning of the COVID-19 pandemic, the health authority has struggled to set up facilities and isolation centers for COVID-19. They also struggled to identify risks and manage resource allocation, which, as a result, distracted from controlling the disease [19]. Although some measures like quarantine and airport closures have been taken, only a few hospitals and medical facilities were equipped to handle COVID-19 cases [19]. Pre-existing weaknesses in disaster preparedness had also been documented before the COVID-19 pandemic [24].In a 2018 study of 531 Yemeni health professionals, 32.0% had good knowledge of emergency and disaster preparedness and 41.0% reported having received no courses in disaster preparedness [24].

Taken together, these findings suggest that pandemic preparedness in Yemen requires coordinated interventions across multiple socio-ecological levels. At the health-system level, preparedness should include continuous workforce training, infection-prevention capacity, basic diagnostic services, availability of essential supplies and clear referral pathways. At the community level, interventions should address misinformation, mistrust, stigma and the affordability of preventive measures. Structural improvements in health-system resilience remain important, but preparedness planning should also identify interventions that can be implemented despite continuing fragility.

Participants in this study perceived shortages of medical resources, inadequate infection-prevention practices, limited staff preparedness and restricted PCR testing as barriers to accessing appropriate care. These perceptions are consistent with previously documented weaknesses in Yemen's health-system capacity during COVID-19 [13,19,21]. Importantly, the present qualitative findings show how such system-level limitations were experienced at the community level: concerns about infection in healthcare facilities, long waiting times, high costs and uncertainty about available services contributed to reluctance to seek formal care.

### Role of community and civil-society actors

Health authorities alone may have limited capacity to address these barriers in a prolonged humanitarian crisis. Community-based organisations, local civil-society groups, community and religious leaders, volunteers and humanitarian organisations may therefore have an important complementary role in supporting risk communication and community engagement [14,25]. Trusted community actors could support two-way risk communication, identify and respond to circulating rumours, reduce stigma and communicate where and how appropriate healthcare can be accessed, and provide feedback on community concerns [16,25]. Humanitarian actors may also support provision of essential preventive supplies, infection-prevention resources and referral mechanisms where public-sector capacity is constrained.

Such engagement should not replace government responsibility for health-system preparedness. Rather, it can provide an additional mechanism for reaching communities, building trust and adapting interventions to local concerns during periods in which institutional capacity is limited.

### Implications for preparedness and policy

The findings support a focused set of preparedness actions rather than broad recommendations that may be difficult to implement in the Yemeni context.

In the short term, local health authorities, community-based organisations, trusted community and religious leaders, and humanitarian partners could support community-based rumour monitoring, culturally adapted risk communication, provision of affordable or free essential preventive supplies, and strengthened communication regarding available health services. These actions could help address misinformation, mistrust and financial barriers [16,25]. In the medium term, health authorities and humanitarian partners should prioritise infection-prevention training, reliable access to personal protective equipment, diagnostic and referral pathways and emergency supply mechanisms. Longer-term preparedness requires integration of these measures into national and local emergency plans, including workforce training, surge capacity, community engagement and mechanisms for maintaining essential services during crises. Recent work from conflict-affected Sudan similarly emphasises the importance of coordinated and adaptable outbreak-response mechanisms when conventional health-system structures are disrupted by conflict [26].

A minimum preparedness package for fragile settings could therefore include trusted risk communication and rumour monitoring; community engagement mechanisms; basic infection-prevention capacity; trained healthcare workers; essential protective and medical supplies; functional diagnostic and referral pathways; and plans for maintaining essential health services during outbreaks. The package should be adapted to existing local capacity and implemented jointly by health authorities, local actors and humanitarian partners. This approach is consistent with recent WHO guidance recommending a prioritised and context-adapted package of essential health services for populations affected by protracted humanitarian emergencies [27,28].

These recommendations are findings-informed rather than direct priorities stated by participants and should therefore be interpreted as practical implications derived from the barriers identified in this study.

### Limitations of the study

This is a qualitative study using in-depth interviews with adult community members in Aden, Yemen for primary data collection. Several limitations should be considered when interpreting the findings. First, the study was conducted in a single governorate, Aden, and participants were selected from individuals who had previously participated in the parent community-based KAP survey. Therefore, individuals who did not participate in the parent survey were not represented in the qualitative sample. The findings therefore reflect the experiences and perceptions of this group and should not be interpreted as statistically representative of the wider population of Aden or Yemen. Second, participants’ accounts were self-reported and may have been affected by recall bias. Participants’ responses may also have been influenced by social-desirability bias, particularly when discussing their own preventive practices or healthcare-seeking behaviours. Third, individuals who had symptoms of COVID-19 or who were isolated or in quarantine at the time of recruitment were not eligible to participate. Consequently, the study did not directly capture the experiences of individuals who were acutely ill or undergoing isolation, whose experiences of healthcare-seeking and access to services may have differed from those of the participants interviewed. Fourth, interviews were conducted in Arabic and subsequently translated into English for analysis.

Although the translated transcripts were reviewed against the original Arabic recordings, some linguistic, cultural or contextual nuances may have been reduced or altered during the translation process. Fifth, as in all qualitative research, interpretation may have been influenced by the researchers’ professional backgrounds and prior knowledge of the Yemeni health system. The involvement of multiple researchers in coding, interpretation and review of the themes was used to reduce reliance on a single perspective; nevertheless, researcher positionality should be considered when interpreting the findings. Finally, the study was not designed to systematically examine differences in experiences according to gender or other sociodemographic characteristics. Therefore, the findings should not be interpreted as demonstrating gender-specific patterns. An additional limitation relates to the use of the SEM. The interview guide was not originally designed around the SEM; rather, the framework was applied after the inductive analysis as an interpretive lens. Consequently, influences at the different SEM levels may not have been elicited with equal depth. Future research could use interview guides explicitly informed by the SEM while retaining sufficient openness to identify inductively emerging factors. Future studies could use multi-site qualitative or mixed methods designs across different governorates and population groups to examine how barriers vary according to gender, socioeconomic position, displacement status, and other dimensions of vulnerability, and to assess the feasibility of preparedness interventions identified in this study.

## Conclusion

This study highlights the multifaceted barriers to COVID-19 prevention and healthcare-seeking in Aden, Yemen. Interpreted through the SEM, participants’ accounts identified barriers operating across five interacting levels: intrapersonal/individual factors including denial, fear, and financial constraints; interpersonal stigma, mockery and social pressure; organisational/institutional limitations in healthcare access, preparedness and service delivery; community-level misinformation and mistrust; and public policy/structural constraints associated with resource limitations, perceived governmental preparedness and prolonged conflict. These interconnected factors were perceived by participants to influence both the adoption of preventive measures and decisions to seek healthcare.

Interpreted through the Socio- Ecological Model, the findings suggest that individual behaviours cannot be understood separately from interpersonal influences, community-level processes, organisational and health-system conditions, and the wider public policy and structural context of prolonged conflict. The use of the local term “Jazaa” further illustrates how culturally embedded interpretations of illness may shape risk perception and responses to an emerging infectious disease. Preparedness for future outbreaks in fragile and conflict-affected settings should therefore combine feasible interventions across these levels, including culturally appropriate and trusted risk communication, community engagement and rumour management, affordable access to preventive resources and healthcare, strengthened infection-prevention capacity, accessible diagnostic and referral pathways, adequate essential medical supplies and continued training of healthcare workers.

Although these measures cannot address the broader political and structural causes of health-system fragility, they may strengthen community trust, improve access to essential services and enhance preparedness for future public health emergencies in settings experiencing prolonged humanitarian crises.

## Supporting information

S1 Appendix Semi-structured interview guide used for in-depth interviews. (PDF)

S1 Checklist Inclusivity in global research questionnaire. (DOCX)

S2 Checklist Consolidated Criteria for Reporting Qualitative Research (COREQ) checklist. (DOCX)

## References

[1] Marshall S, Duryea M, Huang G, Kadioglu O, Mah J, Palomo JM, et al. COVID-19: What do we know?. Am J Orthod Dentofacial Orthop. 2020;158(5):e53–62. doi: 10.1016/j.ajodo.2020.08.010 33131568 PMC7505627

[2] Johns Hopkins University. COVID-19 Dashboard by the Center for Systems Science and Engineering (CSSE). 2023. Accessed 2026 June 1. https://coronavirus.jhu.edu/map.html

[3] Qirbi N, Ismail SA. Health system functionality in a low-income country in the midst of conflict the case of Yemen. Health Policy Plan. 2017;32(6):911–22. 10.1093/heapol/czx031 28402469

[4] Braam DH, Srinivasan S, Church L, Sheikh Z, Jephcott FL, Bukachi S. Lockdowns, lives and livelihoods: The impact of COVID-19 and public health responses to conflict affected populations - a remote qualitative study in Baidoa and Mogadishu, Somalia. Confl Health. 2021;15(1):47. doi: 10.1186/s13031-021-00382-5 34118985 PMC8197606

[5] Mousavi SM, Anjomshoa M. COVID-19 in Yemen: A crisis within crises. Int J Equity Health. 2020;19(1):120. doi: 10.1186/s12939-020-01231-2 32652995 PMC7351642

[6] Elnakib S, Elaraby S, Othman F, BaSaleem H, Abdulghani AlShawafi NA, Saleh Al-Gawfi IA, et al. Providing care under extreme adversity: The impact of the Yemen conflict on the personal and professional lives of health workers. Soc Sci Med. 2021;272:113751. doi: 10.1016/j.socscimed.2021.113751 33588206 PMC7938221

[7] Hassan OEH, Al-Aghbari AA, McGowan M, Dar Iang M, Basaleem HO, Al-Sakkaf KA, et al. Knowledge, attitudes, and practices toward COVID-19 prevention in Yemen: A community-based cross-sectional study. Front Public Health. 2023;11:1178183. doi: 10.3389/fpubh.2023.1178183 37492140 PMC10364124

[8] Kilanowski JF. Breadth of the socio-ecological model. J Agromedicine. 2017;22(4):295–7. doi: 10.1080/1059924X.2017.1358971 28742433

[9] McLeroy KR, Bibeau D, Steckler A, Glanz K. An ecological perspective on health promotion programs. Health Educ Q. 1988;15(4):351–77. doi: 10.1177/109019818801500401 3068205

[10] Graneheim UH, Lundman B. Qualitative content analysis in nursing research: Concepts, procedures and measures to achieve trustworthiness. Nurse Educ Today. 2004;24(2):105–12. doi: 10.1016/j.nedt.2003.10.001 14769454

[11] Elo S, Kyngäs H. The qualitative content analysis process. J Adv Nurs. 2008;62(1):107–15. doi: 10.1111/j.1365-2648.2007.04569.x 18352969

[12] Tong A, Sainsbury P, Craig J. Consolidated criteria for reporting qualitative research (COREQ): A 32-item checklist for interviews and focus groups. Int J Qual Health Care. 2007;19(6):349–57. doi: 10.1093/intqhc/mzm042 17872937

[13] Hyzam D, Boah M, Dhaiban M, Dureab F, Al-Mahbashi T, Hadi A, et al. When formal governance persists but resilience does not: Health system governance and resilience in Yemen across a decade of conflict and crisis (2014-2025). BMC Health Serv Res. 2026;26(1):851. doi: 10.1186/s12913-026-14957-6 42316231 PMC13282849

[14] Bogale B, Scambler S, Mohd Khairuddin AN, Gallagher JE. Health system strengthening in fragile and conflict-affected states: A review of systematic reviews. PLoS One. 2024;19(6):e0305234. doi: 10.1371/journal.pone.0305234 38875266 PMC11178226

[15] Lapinski MK, Oetzel JG, Park S, Williamson AJ. Cultural tailoring and targeting of messages: A systematic literature review. Health Commun. 2025;40(5):808–21. doi: 10.1080/10410236.2024.2369340 38961665

[16] World Health Organization, Regional Office for Europe. Risk communication, community engagement and infodemic management at the core of health security in humanitarian emergencies: operational tool designed from experiences in the WHO European Region. Copenhagen: WHO Regional Office for Europe; 2026.

[17] Ahmed A, Al-Aghbari AA, Hassan OE-H, McGowan M, Al-Qadasi Y, Dureab F. The COVID-19 infodemic as a major driver for vaccine hesitancy in conflict settings: A mixed-method study. Discov Health Systems. 2024;3(1). doi: 10.1007/s44250-024-00127-0

[18] Blukacz A, Obach A, Vásquez P, Campaña C, Huerta C, Bernal Y, et al. Determinants of COVID-19 and non-COVID-19 vaccine confidence in low- and middle-income countries: A systematic review of qualitative evidence and thematic synthesis. Vaccine. 2025;44:126546. doi: 10.1016/j.vaccine.2024.126546 39608248

[19] Alsabri M, Alhadheri A, Alsakkaf LM, Cole J. Conflict and COVID-19 in Yemen: Beyond the humanitarian crisis. Global Health. 2021;17(1):83. doi: 10.1186/s12992-021-00732-1 34294097 PMC8295627

[20] Asadi-Aliabadi M, Tehrani-Banihashemi A, Moradi-Lakeh M. Stigma in COVID-19: A barrier to seek medical care and family support. Med J Islam Repub Iran. 2020;34:98. doi: 10.34171/mjiri.34.98 33315991 PMC7722951

[21] Dureab F, Al-Awlaqi S, Jahn A. COVID-19 in Yemen: Preparedness measures in a fragile state. Lancet Public Health. 2020;5(6):e311. doi: 10.1016/S2468-2667(20)30101-8 32334648 PMC7180012

[22] Kakietek J, Dayton Eberwein J, Kerr A, Stacey N. To what extent did households in developing countries forgo needed healthcare during the COVID-19 pandemic? Repeated survey estimates from 25 countries in 2020 and 2021. BMJ Public Health. 2024;2(2):e001027. doi: 10.1136/bmjph-2024-001027 40018592 PMC11816704

[23] Neyazi N, Mirzazadeh A, Ibrahimi AG, Ahmadzai AM, Tanoli JA. Changes and backlogs in the provision and utilization of essential health services in Afghanistan during and after the COVID-19 pandemic. BMC Health Serv Res. 2025;25(1):923. doi: 10.1186/s12913-025-13093-x 40611256 PMC12232008

[24] Naser WN, Saleem HB. Emergency and disaster management training; knowledge and attitude of Yemeni health professionals- a cross-sectional study. BMC Emerg Med. 2018;18(1):23. doi: 10.1186/s12873-018-0174-5 30081832 PMC6091203

[25] World Health Organization. WHO policy brief: Building trust through risk communication and community engagement and infodemic management. Geneva: World Health Organization; 2024.

[26] Ayyad FA, Izzoddeen A, Elbukhari IM, Awadalla H. Rethinking cholera response: An orchestration framework for future preparedness in fragile conflict-affected settings, a case study from Sudan. Frontiers in Public Health. 2026;14. doi: 10.3389/fpubh.2026.1836433 PMC13364977 42454312

[27] World Health Organization. High-priority health services for humanitarian response (H3 package). Geneva: World Health Organization; 2024.

[28] Griekspoor A, Kampalath VN, Broccoli MC, Fogarty J, Pasha E, Zunong N, et al. The development of the H3 package: A Package of high-priority health services for humanitarian response. BMJ Glob Health. 2026;11(1):e020120. doi: 10.1136/bmjgh-2025-020120 41545213 PMC12815147

