# Supplementary material for: Barriers to prevention and healthcare-seeking in a fragile humanitarian setting: Evidence from Yemen during the COVID-19 pandemic
Source: PLOS Glob Public Health. 2026 Oct 15;6(10):e0007435. doi: 10.1371/journal.pgph.0007435 (PMC13671496; doi:10.1371/journal.pgph.0007435)
Supplement: Supplementary file 1 [file pgph.0007435.s001.pdf]

S1 Appendix. Semi-structured interview guide used for in-depth interviews

Barriers to prevention and healthcare-seeking in a fragile humanitarian setting: Evidence from Yemen during the COVID-19 pandemic

Interview record

Interview date (day-month-year): ______________________________

Location/district: ____

Participant reference number: _________________________________

Interviewer name: ____

Interview duration (minutes): _________________________________

Audio recording completed: ☐ Yes ☐ No

Interview interrupted: ☐ Yes ☐ No

If yes, brief note on interruption(s): _________________________

Respondent information

Age: __________________

Sex: ☐ Female ☐ Male

Marital status: ☐ Married ☐ Other

Education: ____

Occupation: ____

General information for interviewers

This guide was used to support semi-structured in-depth interviews with adult community members in Aden, Yemen. The purpose of the interviews was to explore participants' perceptions and experiences related to COVID-19, preventive practices, healthcare-seeking, and the barriers that influenced these behaviours during the humanitarian crisis. Interviewers could use follow-up probes to clarify responses and explore issues raised by participants in greater depth.

Interviews were conducted in Arabic. Interviewers were instructed to establish rapport, use open-ended questions, remain neutral, avoid leading participants, and allow participants to describe their experiences in their own words.

Introduction to the participant

Good morning/afternoon/evening Mr/Ms/Mrs ____________________.

My name is ____________________. Thank you for taking the time to participate in this interview. We are

conducting interviews with community members in Aden to understand experiences, challenges and barriers related to COVID-19 prevention and healthcare-seeking during the current humanitarian situation in Yemen.

The interview will take approximately 25–35 minutes. There are no right or wrong answers; we are interested in your experiences and views. With your permission, I would like to audio record the interview so that important information is not missed. All information will be treated confidentially and stored securely with access limited to the research team. Your identity will not be included in the analysis or reporting. Participation is voluntary, and you may choose not to answer any question or stop the interview at any time.

Before starting, please confirm:

Do you have any questions about the interview?

Do you agree to participate in the interview?

Have you signed the written informed consent form?

Do you agree to the interview being audio recorded?

Questions for community members

1. Perceptions and experiences of COVID-19

How would you describe the current situation with respect to COVID-19?

What comes to your mind when you hear about COVID-19 or about a person with COVID-19 in your area or household?

How are you dealing with the situation? How do people around you deal with someone who has COVID-19?

Have you been infected with COVID-19 or suspected that you had it? If yes, please tell me about your experience and how people dealt with you.

If you have not been infected, what do you think would happen if you were infected? Do you think COVID-19 is associated with stigma or negative reactions in the community?

2. Preventive practices and barriers

What preventive measures against COVID-19 do you know about or follow?

What do you think about these preventive measures?

What makes it easy or difficult for you or other people in the community to follow preventive measures?

Are there any social, financial, practical or other challenges that affect the use of preventive measures? Please explain.

3. Experiences with healthcare and healthcare-seeking

Can you describe any experience of visiting a health facility during the COVID-19 pandemic and the current humanitarian situation?

What made you visit the health facility?

How were you treated?

Did you face any barriers or challenges during the visit? Please explain.

What challenges might prevent you, or other people in your community, from seeking healthcare when it is needed?

Can you give examples of factors that would make you decide to seek care or not to seek care?

4. Community, health-system and wider contextual influences

If relevant to the participant's responses, explore how community attitudes, information or rumours, trust, healthcare-service conditions, governmental response, financial constraints, and the wider humanitarian or conflict situation influence prevention or healthcare-seeking.

Suggested probes

Can you tell me more?

What do you mean by that?

Can you give me an example?

What happened next?

Why do you think that happened?

How did that affect what you did or decided to do?

Is there anything else you would like to add?

Closing

I do not have any more questions. Thank you very much for sharing your experiences and views with us. Is there anything else you would like to add, or do you have any questions before we finish?

Interviewer notes and reflexive comments

After the interview, record any relevant contextual notes, interruptions, or observations, and reflect briefly on whether the interviewer's presence, wording, or reactions may have influenced the discussion.

____

____

____

____

____

____
