# Supplementary material for: Barriers to prevention and healthcare-seeking in a fragile humanitarian setting: Evidence from Yemen during the COVID-19 pandemic
Source: PLOS Glob Public Health. 2026 Oct 15;6(10):e0007435. doi: 10.1371/journal.pgph.0007435 (PMC13671496; doi:10.1371/journal.pgph.0007435)
Supplement: Supplementary file 3 [file pgph.0007435.s003.docx]

**Consolidated Criteria for Reporting Qualitative Research (COREQ): 32-item checklist**

**Manuscript title:** Barriers to Prevention and Healthcare Seeking in a Fragile Humanitarian Setting: Evidence from Yemen During the COVID-19 Pandemic

**Domain 1: Research Team and Reflexivity**

**1. Interviewer/facilitator :** Which author(s) conducted the interviews?

Data collection was conducted by two research teams, each comprising one research professional and two interviewers. The manuscript does not identify the individual interviewers or specify which interviewers were authors.

**2. Credentials :** What were the researchers’ credentials?

The research team included researchers based in Yemen and at international academic institutions. The wider research team had public-health and qualitative-research experience, while Yemeni researchers contributed knowledge of the local language, culture and health system. Individual interviewer credentials are not reported.

**3. Occupation:** What was their occupation at the time of the study?

Each data-collection team consisted of one research professional and two interviewers. Further occupational details are not reported.

**4. Gender :** Was the researcher male or female?

Not reported.

**5. Experience and training:** What experience or training did the researchers have?

Interviewers received training in qualitative interviewing, including rapport building, open-ended questioning, neutrality, and avoidance of leading questions.

**6. Relationship established:** Was a relationship established before study commencement?

Not reported.

**7. Participant knowledge of the interviewer:** What did participants know about the researcher?

Not specifically reported. Participants received a study information sheet and consent form before providing written informed consent, but the manuscript does not report what participants were told about the individual interviewers or researchers.

**8. Interviewer characteristics:** What characteristics were reported about the interviewer?

The research team combined local contextual knowledge from Yemen with public-health and qualitative-research experience. The manuscript also reports reflexive discussions about how researchers’ professional backgrounds and knowledge of the Yemeni context might influence data collection and interpretation. Emerging findings were reviewed by multiple researchers to challenge assumptions and strengthen interpretation.

**Domain 2: Study Design**

**9. Methodological orientation and theory:** What methodological orientation was stated to underpin the study?

Inductive qualitative content analysis was used. The Socio-Ecological Model was subsequently applied as an interpretive framework; it was not used to impose predetermined codes.

**10. Sampling:** How were participants selected?

Participants were systematically selected from 400 adults who had completed the preceding KAP survey. One participant was randomly selected from among the first 16 participants on the survey list, after which every 16th participant was selected and invited to participate in an in-depth interview.

**11. Method of approach:** How were participants approached?

Selected participants from the KAP survey sampling frame were invited to participate in an in-depth interview. The specific mode of contact or invitation is not reported.

**12. Sample size:** How many participants were in the study?

Twenty-five participants were interviewed and included in the final qualitative analysis. Data saturation was considered to have been reached at the 22nd interview; the remaining three planned interviews were completed and did not generate additional themes.

**13. Non-participation:** How many people refused to participate or dropped out? Reasons?

No invited participants refused participation, and no participants withdrew.

**14. Setting of data collection:** Where was data collected?

Face-to-face interviews were conducted in participants’ households in Aden, Yemen.

**15. Presence of non-participants:** Was anyone else present besides participants and researchers?

Not reported.

**16. Description of sample:** What are the important characteristics of the sample?

Participants were adult residents of Aden aged 18 years or older. Individuals with COVID-19 symptoms or who were isolated or in quarantine at the time of data collection were excluded. Sociodemographic characteristics are presented in Table 1.

**17. Interview guide:**Were questions, prompts or guides provided? Was it pilot tested?

A semi-structured in-depth interview guide was developed specifically for the study and is provided as S1 Appendix. It covered perceptions and beliefs regarding COVID-19, preventive practices and barriers, healthcare-seeking experiences, governmental and structural barriers, and experiences of healthcare services. The guide was reviewed by the research team and relevant experts and was pilot-tested with community members who had sociodemographic characteristics similar to the study population. Two questions were revised for clarity and sequencing before formal data collection. The core domains remained unchanged during data collection.

**18. Repeat interviews:** Were repeat interviews carried out?

No repeat interviews are reported.

**19. Audio/visual recording:** Did the research use audio or visual recording?

Yes. All interviews were audio-recorded with participants’ consent.

**20. Field notes:** Were field notes made during and/or after interviews?

Interview notes were securely stored together with the interview audio recordings. The manuscript does not specify whether formal field notes were systematically made during or immediately after each interview.

**21. Duration:** What was the duration of the interviews?

Interviews lasted approximately 25–35 minutes.

**22. Data saturation:** Was data saturation discussed?

Yes. Saturation was assessed during concurrent data collection and preliminary analysis by the principal investigator and two researchers from Heidelberg University. It was considered to have been reached at the 22nd interview, when no new themes, insights or substantively new information relevant to the study objectives emerged. The remaining three planned interviews were completed and did not generate additional themes, supporting the assessment of saturation.

**23. Transcripts returned:** Were transcripts returned to participants for comment or correction?

Not reported.

**Domain 3: Analysis and Findings**

**24. Number of data coders:** How many data coders coded the data?

Two research professionals independently conducted the initial coding and theme development. The emerging codes, sub-themes and overarching themes were subsequently reviewed by two principal researchers. Differences in coding or interpretation were discussed until consensus was reached.

**25. Description of the coding tree:** Did authors describe the coding tree?

Yes. An analytic coding tree was developed to organise the relationships between codes, sub-themes and overarching themes in relation to the study objectives.

**26. Derivation of themes:** Were themes identified in advance or derived from the data?

Themes were derived inductively from the data. The Socio-Ecological Model was applied only after the inductive analysis as an interpretive framework.

**27. Software:** What software was used to manage the data?

NVivo 12 was used for qualitative data management and inductive coding

**28. Participant checking:** Did participants provide feedback on the findings?

Formal participant checking of the final findings is not reported. During the interviews, participants were given opportunities to clarify or confirm the interviewer’s understanding of their responses to help ensure that their intended meanings were accurately understood.

**29. Quotations presented:** Were participant quotations presented to illustrate themes/findings? Was each quotation identified?

Yes. Representative participant quotations are presented throughout the Results section and tables and are identified using anonymised informant numbers.

**30. Data and findings consistent:** Was there consistency between the data presented and the findings?

Yes. The reported themes are supported by representative participant quotations.

**31. Clarity of major themes:** Were major themes clearly presented in the findings?

Yes. The identified barriers are presented across five SEM levels: intrapersonal/individual, interpersonal, organisational/institutional, community, and public policy/structural, with an additional section describing interconnections across levels.

**32. Clarity of minor themes/diverse cases:** Is there a description of diverse cases or discussion of minor themes?

Yes. The Results describe variation in participants’ experiences and perceptions, including differing levels of awareness, preventive behaviour, trust, and healthcare-seeking experiences.
